# A new circle method for measuring humeral torsion on MRI-scans less sensitive to Hill-Sachs lesions

**DOI:** 10.1016/j.ejro.2022.100468

**Published:** 2022-12-14

**Authors:** Stefan Demarmels, Holger Grehn, Dirk Müller, Andreas U. Freiburghaus, Arno Frigg

**Affiliations:** a Department of Orthopaedic and Trauma Surgery, Kantonsspital Graubünden, Chur, Switzerland; b Department of Radiology, Kantonsspital Graubünden, Chur, Switzerland; c University of Zürich, Zürich, Switzerland; d Department of Orthopaedic Surgery, University Hospital Basel, Basel, Switzerland

**Keywords:** HTA, humeral torsion angle, ICC, Intra Class Correlation, Humeral torsion angles, Methodology, MRI, CT

## Abstract

**Objectives:**

The literature on humeral torsion angles (retrotorsion) reveals great inconsistencies between methodology and values. Decreased retrotorsion was suspected to correlate with instability, but evidence is contradictory. The measurement according to the gold standard method of Bernageau and Godefroy (B&G) can be challenging especially in the presence of Hill-Sachs-lesions. Therefore, we have developed and evaluated a new measurement method for the humeral torsion angle on MRI-scans.

**Materials and Methods:**

Three investigators have measured 67 patients (35 with shoulder instability, 32 healthy) on axial MRIs with 603 measurements used for reliability calculation. The new Circle-method determines the retrotorsion by overlaying two circles on the transversal section of the humeral head. The first circle is adjusted congruent with the margin of the humeral head, whereas the second circle is adjusted to the greater tubercle. The line bisecting the centres of these circles is defined as the humeral head axis. This method was compared to B&G.

**Results:**

The mean retrotorsion angle of all patients was 25°± 25° (mean ± SD) with B&G, and 24° ± 27° with the Circle-method. Neither method revealed a significant difference between stable and unstable shoulders (p = 0.47). Of the 35 patients with unstable shoulders 21 (60%) presented Hill-Sachs lesions. No significant differences between patients with or without Hill-Sachs lesions (Circle-method: p = 0.61; B&G: p = 0.67). The reliability parameters for both methods were similar.

**Conclusions:**

The new Circle-method is as precise as the method of B&G. It may yield more consistent values in cases with substantial Hill-Sachs-lesions. Our data do not suggest retrotorsion as a predictor of instability.

## 1 Introduction

Predisposing factors for shoulder instability and (sub)luxation have repeatedly been investigated. One of the anatomical factors considered is humeral torsion (or retrotorsion). It is defined as the angle between the humeral head axis and the epicondylar axis (humeral torsion angle, HTA). In the resting, non-abducted position of the arm the humeral head points towards postero-medial. The commonly reported position of the arm for anterior dislocations is 90° abduction and external rotation. In this position the humeral head points towards anterior. If in such a position the humeral retrotorsion is diminished, the threshold for a spontaneous dislocation of the shoulder may be lowered, i.e., subluxation may occur earlier at lower degrees of abduction and/or external rotation [1]. Reviewing 40 methodologically defined studies published in the last 108 years [2], [3], [4], [5], [6], [7], [8], [9], [10], [11], [12], [13], [14], [15], [16], [17], [18], [19], [20], [21], [22], [23], [24], [25], [26], [27], [28], [29], [30], [31], [32], [33], [34], [35], [36], [37], [38], [39], [40] we found greatly varying methods and results. Most reports have investigated between 3 and 250 individuals, only one study has investigated 410 persons (Table 1). Reported humeral torsion angle measurements were performed on specimens, X-rays, CT-scans, ultrasound-scans or MRI-scans, and results ranged between 4° and 76° with large standard deviations. These reported means vary greatly between methods, authors, and source (imaging/cadaver), and the deviations are large. The definition of a "normal" value and range for the humeral torsion is thus impossible and for practical clinical purposes non-existent.

**Table 1:** Literature overview of humeral torsion angles (HTA).

ex	Author	n (measured humeri)	Average humeral retrotorsion angle
			(range or ± SD); degrees
**Methods using the measurement technique according to Bernageau/Godefroy**[42]**with CT or MRI scans**			
^1^	Bernageau[42]	N/A	N/A (only description of measuring method with CT)
^2^	Matsumura[22]	410	26° ( ± 11)
^3^	Cassagnaud[4]	64	11.71° (−17.5°−54°) dominant
			7.03° (−28°−47°) non-dominant
^4^	Symeonides[36]	80	16.1° ( ± 11.07) stable group
			4.3° ( ± 10.56) unstable group
^5^	Randelli[29]	180	30° (25°−35°) stable group
			30° (28°−35°) unstable group
^6^	Laumann[21]	32	28°− 31°
^7^	Oh[24]	28	31.42° ( ± 12.1)
^8^	Tellioğlu[37]	36	19.5° (−4 to 41°)
^9^	Myers[23]	24	32.4° ( ± 11.4) dominant
			25.2° ( ± 7.7) non-dominant
^10^	Pan[26]	20 cadaveric specimens	32.1° ( ± 14.1)
^11^	Guenoun[12]	60 cadaveric specimens	12.3° ( ± 7.9)
^12^	Hernigou[16]	60 cadaveric specimens	23° (15–38)
^13^	Hernigou[17]	120 cadaveric specimens	17.1° ( ± 8.1)
^14^	Boileau[3]	65 cadaveric specimens	16.1° ( ± 13.3)
^15^	Doyle[11]	41 living shoulders and 9 cadaveric specimens	26.8° ( ± 12.2)
**Methods using measurement techniques with CT scans differing from Bernageau/Godefroy**			
^16^	Oh[24]	28	29.7° ( ± 11.66)
			30.64° ( ± 11.24)
			30.41° ( ± 11.17)
			32.14° ( ± 11.7)
^17^	Myers[23]	24	68.3° ( ± 14.2) dominant
			52.5° ( ± 12.6) non-dominant
^18^	Hernigou[16]	60 cadaveric specimens	38° (30°−53°)
^19^	Chu[5]	28	41.1° ( ± 17.1)
^20^	Raniga[30]	59 living shoulders vs. 59 cadaveric specimens	36° ( ± 12) normal group
			14° ( ± 9) Walch type B group
^21^	Saka[34]	28	N/A but reliable method between different testers
^22^	Robertson[32]	60 cadaveric specimens	19° ( ± 6)
^23^	Dähnert[7]	50	55.6° right, 54.6° left
			(values within 56° of difference)
**Methods using measurement techniques with ultrasound**			
^24^	Myers[23]	24	74.2° ( ± 14.5) dominant
			61.2° ( ± 14.4) non-dominant
^25^	Yaari[39]	40	20° ( ± 10) dominant
			29° ( ± 12) non-dominant
^26^	Hannah[13]	30	64.4° ( ± 9.5) measured at sulcus site
			63.1° ( ± 9.6) measured at forearm site
^27^	Dashottar[8]	49	31.5° ( ± 7.5)
^28^	Yoshida[40]	74	68.5° ( ± 10) dominant
			58° ( ± 8.4) non-dominant
^29^	Whiteley[38]	102	18.2° ( ± 9.6) dominant
			19.8° ( ± 10.8) non-dominant
^30^	Ito[18]	58	15.1° ( ± 3.9) right
			15.1° ( ± 2.9) left
^31^	Harland[14]	111	60.9° (85.6% of all values between 40°−80°)
**Methods using measurement techniques with X-ray**			
^32^	Hernigou[17]	120 cadaveric specimens	19.2° ( ± 9.5)
^33^	Boileau[3]	65 cadaveric specimens	22.2° ( ± 14.9)
^34^	Oztuna[25]	40	26° (7°−47°)
^35^	Kronberg[19]	100	33° ( ± 9.3) dominant
			29° ( ± 8.4) non-dominant
^36^	Söderlund[35]	3 cadaveric specimens and 32 living shoulders	N/A (only description of new X-ray method)
^37^	Pieper[28]	175	40.1° ( ± 5.7) normal group
			24.3° ( ± 10.6) anterior dislocation group
			55.7° ( ± 5.1) posterior dislocation group
^38^	Cyprien[6]	158	Stable group:
			22.2° ( ± 10.25) right, 18° ( ± 9.41) left
			Unstable group 1:
			18.5° ( ± 10.39) right, 12.9° ( ± 11.18) left
			Unstable group 2:
			18.2° ( ± 8.12) right, 18.3° ( ± 11.51) left
^39^	Saha[33]	N/A	30° (N/A)
^40^	Debevoise[9]	66	61.2° (47°−85°) stable
			76.5° (63°−104°) unstable
**Methods using measurement techniques with digitization, surface measurements with laser or coordinate machines, palpation, photography, or direct measurements with anatomical landmarks**			
^41^	Boileau[3]	65 cadaveric specimens	17.9° ( ± 13.7) for transepicondylar axis
			21.5° ( ± 15.1) for capitellotrochlear joint line
			17.2° ( ± 12.6) direct measurement
^42^	Harrold[15]	24 cadaveric specimens	18.6° ( ± 10)
^43^	Yaari[39]	40	32° ( ± 6) dominant
			27° ( ± 4) non-dominant
^44^	Patil[27]	250 cadaveric specimens	64.57° ( ± 7.56)
^45^	Dashottar[8]	49	30.5° ( ± 7.9)
^46^	DeLude[10]	28 cadaveric specimens	41.1° ( ± 7.8) left
			35.6° ( ± 9.1) right
^47^	Kummer[20]	420 cadaveric specimens	28.3° ( ± 13.2)
^48^	Roberts[31]	39 cadaveric specimens	21.4° (18.5°−25°)
^49^	Martin[2]	N/A	16° (N/A)

In reconstructive surgery it is essential to measure the humeral torsion either on the affected or also on the contralateral side as reference value [41]. Today this is mostly done in CT-scans but in many institutions MRI-scans are more frequently used as diagnostic tool for evaluation of shoulder pathologies. In the current literature there are only two publications describing a humeral torsion measuring method for MRI-scans. To determine the humeral head axis Doyle et al. [11] and Tellioğlu et al. [37] used a perpendicular line to the anatomical neck between the end of the cartilage on both sides of the humeral head, similar to the gold standard of Bernageau and Godefroy [42]. These measurements were performed in transverse section images positioned at the upper margin of the subscapular tendon. The HTA was the difference between the humeral head axis and the epicondylar axis of the elbow, a line through the largest osseous extension in the transepicondylar section. Determining the exact position of the anatomical neck can sometimes be challenging, especially in the presence of Hill-Sachs-lesions [43], which are frequently seen after shoulder dislocations. These lesions are usually located in the region of the posterior anatomical neck and the point of interest for the axis measurement can be vague. Therefore, we developed a new method using tools available in 3D-viewers (e.g. OsiriX by Pixmeo SARL, Geneva, Switzerland) named “Circle-method”. In the current study we evaluated if (1) the HTA values obtained by the Circle-method and by the method of Bernageau and Godefroy (B&G-method) [42] are comparable, (2) the interobserver variability is similar in both methods, and if (3) in the presence of Hill-Sachs lesions the Circle-method is superior to the B&G-method.

## 2 Materials and methods

### 2.1 Patients

The study was approved by the local Ethical Committee and was conducted in compliance with applicable laws and Good Clinical Practice (GCP, Declaration of Helsinki [44]). Informed consent was obtained from all participants included in the study. 174 patients with clinical shoulder symptoms presenting at our hospital between 2011 and 2014 have undergone MRI examinations of the shoulder. They were allocated to the unstable group or control group. Inclusion criterion for the unstable group was at least one shoulder dislocation, for the control group a stable shoulder. The exclusion criteria for both groups were glenohumeral osteoarthritis of grade 2–4 [45] and/or serious rotator cuff lesions. Additional exclusion criteria for the control group were any shoulder dislocation or subluxation in the history.

Finally, 67 patients, 20 (30%) female and 47 (70%) male, were eligible with a mean age of 43 ± 17 years. The unstable group consisted of 35 (52%) patients, female 10 (29%) and male 25 (71%), with a mean age of 37 ± 19 years. The control group consisted of 32 patients (48%), female 10 (31%) and male 22 (69%), with a mean age of 48 ± 12 years. There was no difference in the gender distribution between groups (p = 0.81). However, the age difference between groups was statistically significant, with the unstable group being on average 11 years younger than control group (p = 0.006).

Osteoarthritis classification was done according to Kellgren and Lawrence [45]. Grade 2 was defined by the presence of a reduced joint line and osteophytes, grade 3 by the additional presence of cysts, and grade 4 by the absence of a joint line and the presence of osteophytes and cysts, or ankylosis. A serious rotator cuff lesion was defined according to Patte [46] (grade 2 or higher) by the presence of a complete rupture of a single rotator cuff tendon with retraction beyond the middle of the humeral head, or the presence of at least two ruptured rotator cuff tendons.

### 2.2 Imaging

All images were acquired using a Philips Achieva 3.0T TX MRI scanner (Philips Healthcare, Best, The Netherlands). The patients were placed in a supine position with arms located by their sides, palmar surfaces facing upwards and shoulders and arms immobile. The axial MR images of the shoulder, which were roughly perpendicular to the humeral shaft axis, were obtained using a dedicated 8-channel shoulder coil using a transverse proton weighted spin echo sequence (TR/TE: 3379/30, field of view: 16 cm, matrix: 380 × 273, number of excitations: 2, slice thickness/gap: 2.5/2.75 mm). In addition, a transverse T1 weighted spin echo sequence of the distal humerus was acquired using the body coil of the MRI scanner (TR/TE: 694/8.8, field of view: 16 cm, matrix: 308 × 240, number of excitations: 2, slice thickness/gap: 2.5/2.75 mm).

### 2.3 HTA measurement

**Fig. 1:**
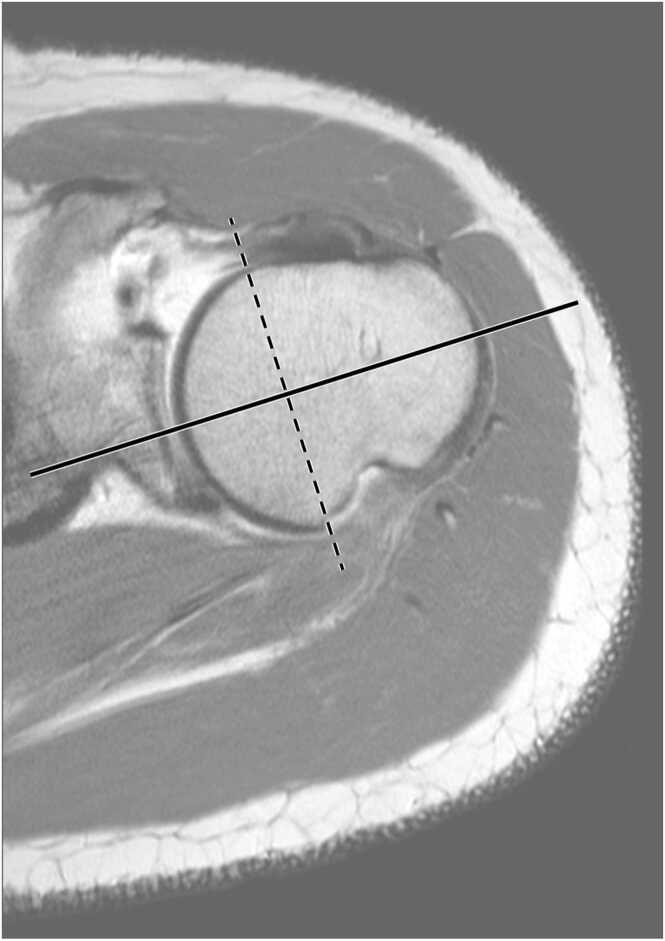
Determination of the humeral head axis on MRI sections by Bernageau and Godefroy’s method [12], [42] in a case with a Hill-Sachs lesion.

**Fig. 2:**
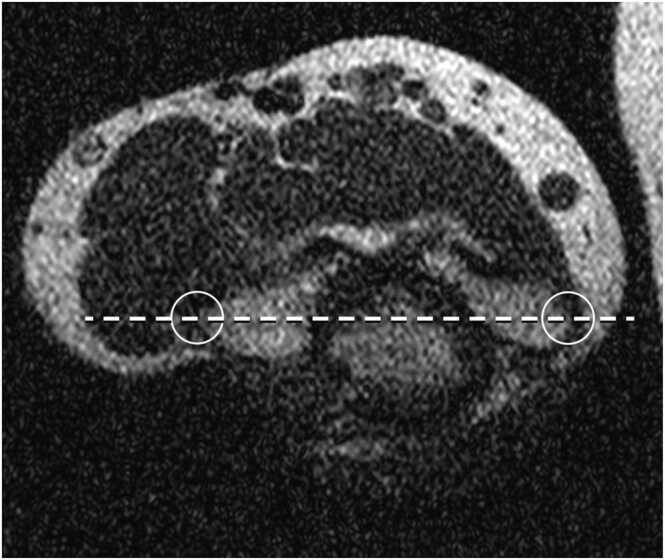
Determination of the epicondylar axis on routine low resolution MRI images.

Measurements were performed with the appropriate tools of the 3D-viewer of the application OsiriX MD (Pixmeo SARL, Geneva, Switzerland) for Mac OS (Apple Inc., Cupertino, USA). The 3D-viewer allows for individual adjustments of the measurement axes. B&G-method was performed according to Bernageau and Godefroy [42], also described in Guenoun et al. [12]. A straight line was drawn intersecting the anterior and posterior limits of the cartilage, corresponding to the anatomical neck at the transverse section image of the humeral head located at the upper margin of the subscapular tendon. The line perpendicular to that line was defined as the humeral head axis (Fig. 1). The HTA was determined as the angle between the humeral head axis and the transepicondylar line as determined in routine low resolution transversal section images (Fig. 2).

**Fig. 3:**
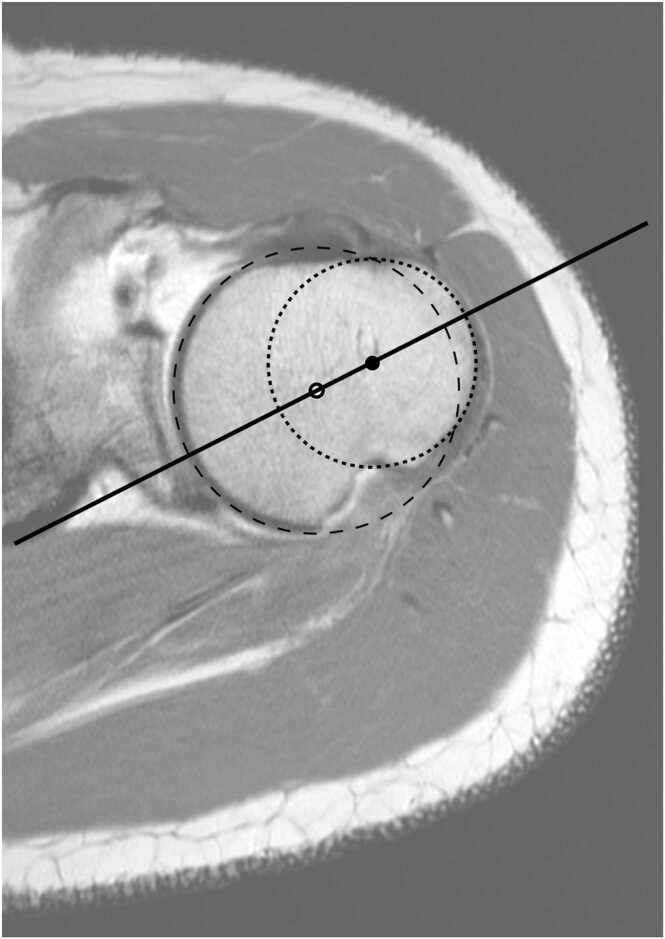
Determination of the humeral head axis by the Circle-method in a case with a Hill-Sachs lesion.

For the Circle-method the axis of the humeral head was determined by overlaying two circles on the image of the transversal section of the humeral head (Fig. 3). The diameter and position of the first circle was adjusted to be congruent with the margin of the humeral head, whereas the second circle was adjusted to be congruent with the margin of the greater tubercle. The line through the centres of both circles was defined as the humeral head axis. The HTA was defined as the angle between the humeral head axis and the transepicondylar line.

In both methods positive HTA values were defined as retrotorsion and negative values as antetorsion. Three investigators proficient in musculoskeletal radiology measured all images after a 50 patients-training phase. They were blinded to the patient’s group and the other observers’ results.

### 2.4 Statistics

Statistical analyses were performed in the R programming language (version 3.3.3, R Core Team, 2017) or with JASP (Version 0.14.1, JASP Team, 2020) [47]. Mean, median and standard deviation (SD) or confidence intervals (CI) were reported. Intraclass correlation coefficients (Cronbach's α [48]) were calculated for the measured parameters. Continuous data was compared with unpaired and paired t-tests; ordinal data was compared with Chi square test. P-values below 0.05 were considered significant. Combined means were calculated from the literature with StatTools [49].

## 3 Results

### 3.1 Measurements

The mean HTA of all 67 patients was 25°± 25° when measured according to Bernageau and Godefroy [41] and 24° ± 27° with the Circle-method. The difference between the methods was not significant (p = 0.57, Table 2). The HTAs of patients with unstable shoulders were not different between methods (p = 0.26, B&G: 24° ± 28; Circle-method: 27° ± 30°, Table 2). The HTAs of patients with stable shoulders showed a significant difference between methods (p = 0.004; B&G: 26° ± 22°; Circle-method: 21° ± 24°; Table 2).

**Table 2:** Humeral torsion angle statistics by method for stable/unstable shoulders: B&G-method [42] vs. Circle-method.

Groups		p	n	Mean (deg)	min. (deg)	max. (deg)	SD (deg)
all	Circle-method	0.57	67	24	-73	83	27
	B&G-method		67	25	-64	80	25
Stable	Circle-method	0.004	32	21	-24	58	24
	B&G-method		32	26	-21	67	22
Unstable	Circle-method	0.26	35	27	-73	83	30
	B&G-method		35	24	-64	80	28
Circle-method	Stable	0.47	32	21	-24	58	24
	Unstable		35	27	-73	83	30
B&G-method, control	Stable	0.74	32	26	-21	67	22
	Unstable		35	24	-64	80	28

### 3.2 Interobserver variability

All 3 investigators have measured the humeral head axis angles with both methods as well as the epicondylar angles in all 67 patients, i.e. 603 measurements were used for reliability calculation. The ICC between 3 investigators over all patients was for the humeral head axis 0.85 with B&G-method and Circle-method and for the epicondylar angle 0.87 (Confidence Interval 0.80–0.92), indicating an excellent agreement. The agreement for the HTA measurements was excellent for both methods with an ICC of 0.98 (Confidence Interval 0.98–0.99).

### 3.3 Hill Sachs lesions

Of the 35 patients with unstable shoulders 21 (60%) presented a Hill-Sachs lesions. The HTAs of these patients showed no difference between the methods (p = 0.13, B&G: 23° ± 28°; Circle-method: 26° ± 32°; Table 3). The Circle-method revealed no statistically significant difference between patients with Hill-Sachs lesion and those without (p = 0.61; no Hill-Sachs: 28° ± 29°; with Hill-Sachs: 26° ± 32°, Table 2). No difference either was found with B&G-method (p = 0. 67; no Hill-Sachs: 26° ± 30°; with Hill-Sachs: 22° ± 28°, Table 3).

**Table 3:** Humeral torsion angle statistics by method for shoulders with/without Hill-Sachs lesions: B&G-method [42] vs. Circle-method.

Groups		p	n	Mean (deg)	min. (deg)	max. (deg)	SD (deg)
No Hill-Sachs lesion	Circle-method	0.75	14	28	-27	83	29
	B&G-method		14	26	-18	80	30
With Hill- Sachs lesion	Circle-method	0.13	21	26	-74	71	32
	B&G-method		21	23	-64	66	28
Circle-method	No Hill-Sachs lesion	0.61	14	28	-27	83	29
	With Hill-Sachs lesion		21	26	-73	71	32
B&G-method	No Hill-Sachs lesion	0.67	14	26	-18	80	30
	With Hill-Sachs lesion		21	22	-64	66	28

### 3.4 Shoulder Instability

The Circle-method revealed no statistically significant difference between stable and unstable shoulders (p = 0.47; stable: 21° ± 24°, n = 32; unstable: 27° ± 30°, n = 35, Table 2). No difference either with B&G-method (p = 0. 47; stable: 26° ± 22°, n = 32; unstable: 24° ± 28°, n = 35; Table 1).

## 4 Discussion

While the B&G-method is well established and frequently used with CT- and MRI-sections, it implies uncertainties in cases where the edges of the articular surface are unclear, e.g. Hill-Sachs-lesions [43]. Therefore, we have developed and evaluated a new measurement method for the humeral torsion angle on MRI-scans. The Circle-Method was found to be as precise as the gold standard method of Bernageau and Godefroy but seemed easier to apply. However, because the observers in this study were very experienced and the training phase consisted of 50 patients, no statistical difference was finally found between the methods in patients with or without Hill-Sachs lesions.

The following considerations have led to suppose that the Circle-method might give less ambiguous measurements than the B&G-method in cases with Hill-Sachs lesions: visually aligning an arc with an inherently circular joint line is more reliable than intersecting a line with two not distinctively unique landmarks as in B&G-method, particularly when lesions obscure the targeted landmarks. Furthermore, at least three points define an arc, whereas only two points are needed to define a line. There are many clouds of representative points available along e.g. the joint line that are known to be connected and lying on an arc. The goodness of fit of the arc is thus more tolerant to erratical or missing individual points.

Next, a drawback of the B&G-method is its strong dependence of position of the axial section plane used to make the measurements. The position of the anterior and posterior edge of the cartilage varies with the cranio-caudal position of the section plane. As a rule, the measurements are made in the axial section plane through the upper limit of the subscapular tendon, marking the upper limit of the lesser tubercle. The Circle-method yields equivalent HTAs in a greater range of sections due to its independence of exact anteroposterior measurement points.

For both methods, the determination of the transepicondylar axis is associated with a considerable measurement error due to the low quality of the scout sections. Moreover, as the sections through the epicondyles are often paracoronary, serial measurements are needed to identify the epicondyles and determine the axis. This compound uncertainty of the epicondylar axis is equal in both the B&G- and the Circle-method and contributes considerably to the great overall variability in HTA values. Furthermore, both modalities are subject to the error caused by movements of the elbow during the scan.

To find a normal value of the HTA, we were reviewing the literature (Table 1). Of the 17 reviewed publications with sufficient number of measurements (number of shoulders and means) the combined means were calculated of measurements in CT-images [5], [7], [22], [23], [24], [30], [36], in radiographs [6], [19], [28] and by ultrasonic scans [8], [13], [18], [23], [38], [39], [40]. The combined mean HTAs were 29° ± 25° (mean ± SD, n = 993), 27° ± 16° (n = 1673) and 36° ± 24° (n = 705), respectively. The results of this study (24° ± 27° with Circle-method and 25° ± 25° with B&G [42]) are somewhat lower than the combined means derived from CT-scans or X-rays, but with very similar variances (Table 1). This range is wide, and therefore, it is impossible to define a normal value for the HTA. It is conceivable that the true biological diversity of HTAs is overlayed by a methodological variability, as in the MRI examination the epicondylar and the shoulder images are acquired with a time delay. Even when the patient does not move, a reposition of the arm cannot always be excluded.

The means for all subgroups were statistically not significantly different between methods, except for the stable shoulders. The Circle-method gave 5° lower angles than B&G-method for stable shoulders, with comparable standard deviations. There is no explanation for this only difference in the current study. Both methods, however, did not reveal statistically significant differences between the HTAs of patients with stable or unstable shoulders. Thus, neither the B&G-method nor the Circle-method could demonstrate the HTA to be a predictor of shoulder instability. This is in accordance with the literature which is contradictory regarding the correlation between diminished retrotorsion and joint dislocation. One group has claimed decreased retrotorsion to be a factor in recurrent shoulder dislocations, as well as dislocations after surgical correction [41]. However, our data do not support recommendations for a surgical torsion correction.

In the group with unstable shoulders 60% of patients showed Hill-Sachs-lesions. While we initially expected to see a narrower standard deviation for the Circle-method than for the B&G-method, we found them to be comparable. An explanation could be the experienced investigators who had performed the B&G-method for years as well as a very long training phase of 50 patients.

## 5 Conclusion

The Circle-method describes a new measurement technique for the humeral torsion. It was found to be as precise as the gold standard method of Bernageau and Godefroy but easier to apply. However, because the observers in this study were very experienced, no statistical difference was found in patients with or without Hill-Sachs lesions. Nonetheless, we hypothesize that the Circle-method could yield more constant values in cases with substantial Hill-Sachs lesions and less experienced observers. Further research is needed into whether beginners would achieve more consistent values with the Circle-method than with the B&G method.

## Ethical statement

Approval from the Institutional Review Board: 17.9.2007.

## Funding

None to declare.

## CRediT authorship contribution statement

**Stefan Demarmels:** Investigation, Formal analysis, Methodology, Validation, Writing – original draft, Writing – review & editing. **Holger Grehn:** Investigation, Methodology, Validation. **Dirk Müller:** Investigation, Methodology, Validation. **Andreas U. Freiburghaus:** Formal analysis, Visualization, Writing – original draft, Writing – review & editing. **Arno Frigg:** Investigation, Conceptualization, Formal analysis, Methodology, Project administration, Validation, Writing – original draft, Writing – review & editing.

## Level of evidence

Diagnostic Studies Level 3.

## Declaration of Competing Interest

The authors declare that they have no known competing financial interests or personal relationships that could have appeared to influence the work reported in this paper.
